# Obstructive Thrombosis of Transcatheter Pulmonary Valve-in-Valve Prosthesis

**DOI:** 10.1016/j.jaccas.2024.102749

**Published:** 2024-12-04

**Authors:** Hanad Bashir, A. Walker Boyd, James G. Jollis, Puvi Seshiah

**Affiliations:** Department of Cardiovascular Medicine, The Christ Hospital, Cincinnati, Ohio, USA

**Keywords:** bioprosthetic pulmonic valve, pulmonic stenosis, transcatheter pulmonary valve replacement, valve thrombosis

## Abstract

A 79-year-old woman, previously treated for congenital pulmonary valve stenosis through surgical and transcatheter procedures, presented with worsening dyspnea. This led to the identification of valve thrombosis in her bioprosthetic pulmonary valve. This report delves into the clinical importance of obstructive valve thrombosis, its diagnostic assessment, and potential therapeutic strategies. This is the first documented case of valve-in-valve thrombosis involving the transcatheter pulmonary valve.

## Graphical Abstract

**Figure:**
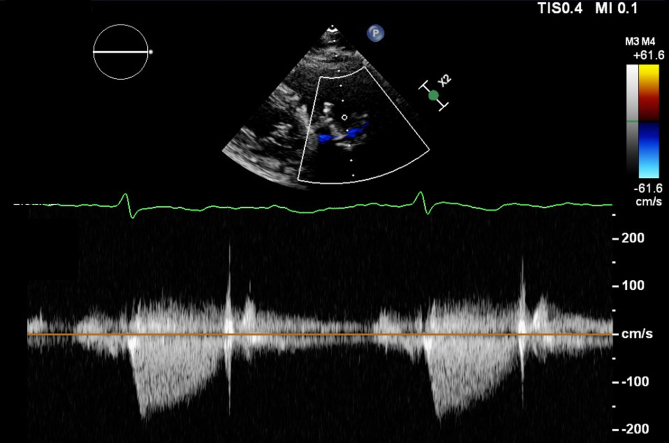

## History of Presentation

A 79-year-old woman with a history of congenital pulmonic valve stenosis, who had a pulmonic valvotomy in childhood and underwent surgical valve replacement in 2006 with a 29-mm Edwards CE Perimount Model 2800 valve (Edwards Lifesciences), developed degeneration of the surgical bioprosthesis with severe pulmonary insufficiency in 2020. She subsequently underwent transcatheter pulmonic valve replacement (TPVR) through a transfemoral approach with a 29-mm Edwards Sapien 3 valve in December 2020. In 2023, she began to experience progressively worsening dyspnea on exertion and lower extremity edema.

**Learning Objectives**

• To be able to identify valve thrombosis on bioprosthetic valves by using multimodal imaging.
• To understand the clinical manifestations and complications of valve thrombosis in bioprosthetic valves.

## Past Medical History

She had a history of congenital pulmonary insufficiency and pulmonic stenosis, necessitating previous surgical and transcatheter replacements, in addition to hypertension, hyperlipidemia, and symptomatic premature ventricular complexes.

## Differential Diagnosis

Differential diagnoses included pulmonary hypertension, hypoattenuating leaflet thickening (HALT), valve thrombosis, acute decompensated heart failure, and prosthesis-patient mismatch from the bioprosthetic pulmonary valve.

## Investigations

**Figure 1:**
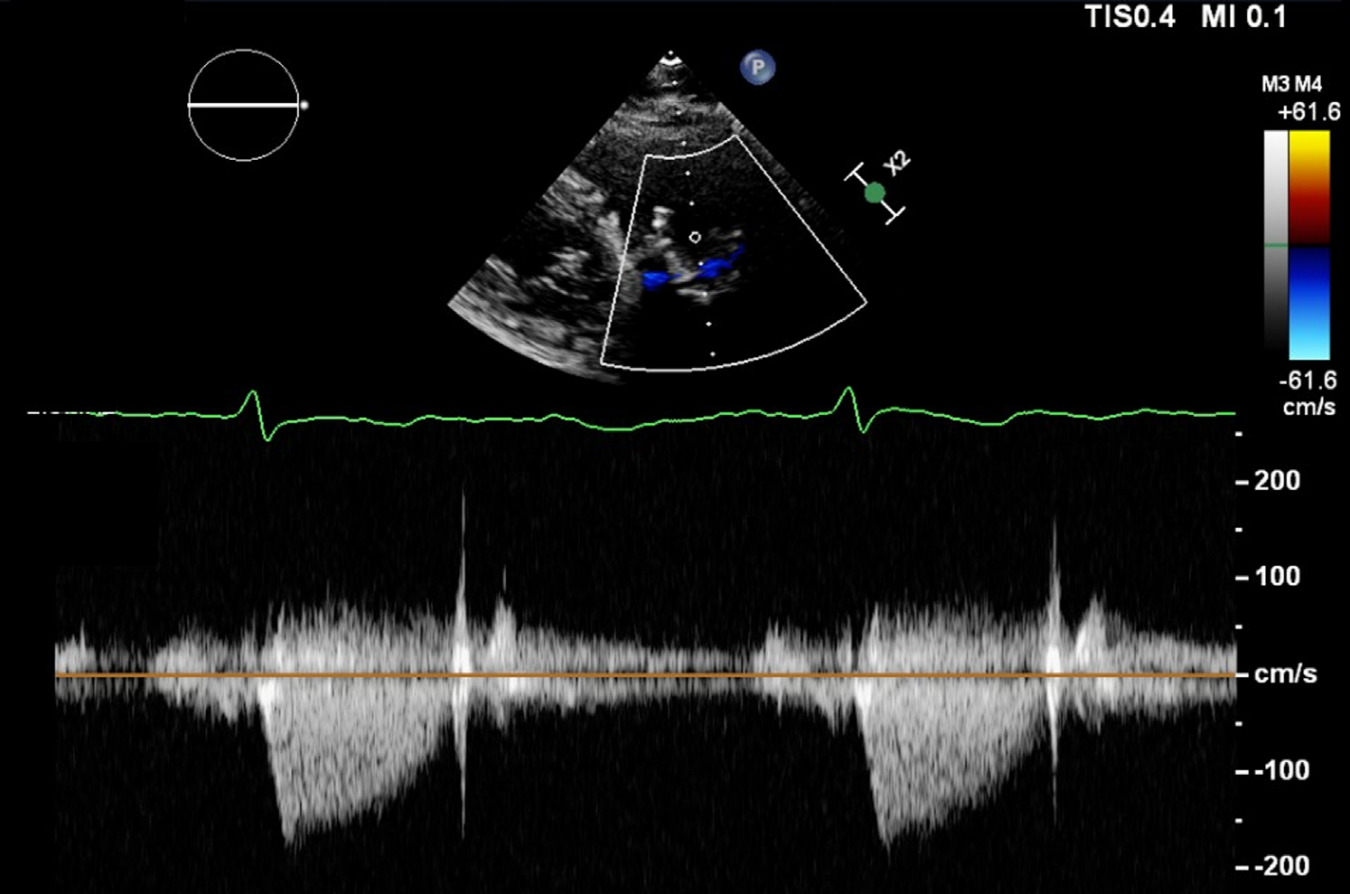
Transthoracic Echocardiogram Continuous Wave Doppler Across the Pulmonary Valve Imaging reveals a mean gradient of 6.1 mm Hg.

In 2020, she underwent cardiac magnetic resonance, cardiac catheterization, cardiac computed tomography angiography (CTA), and transthoracic echocardiography (TTE). These investigations revealed a prosthetic pulmonary valve with moderate to severe insufficiency and mild stenosis, along with elevated right-sided filling pressures, indicating the presence of pulmonary hypertension. The patient underwent TPVR to alleviate her symptoms. The procedure was successful, resulting in symptom relief and restoration of normal biventricular function and filling pressures. The postprocedure mean gradient was 6.1 mm Hg (Figure 1, Video 1). She was started on warfarin for 6 months. Repeat TTE, performed several months later, showed excellent function of the transcatheter pulmonary valve, and the patient’s overall condition appeared stable. Three months after TPVR, she had CTA, which revealed obstructive valve thrombosis.

**Figure 2:**
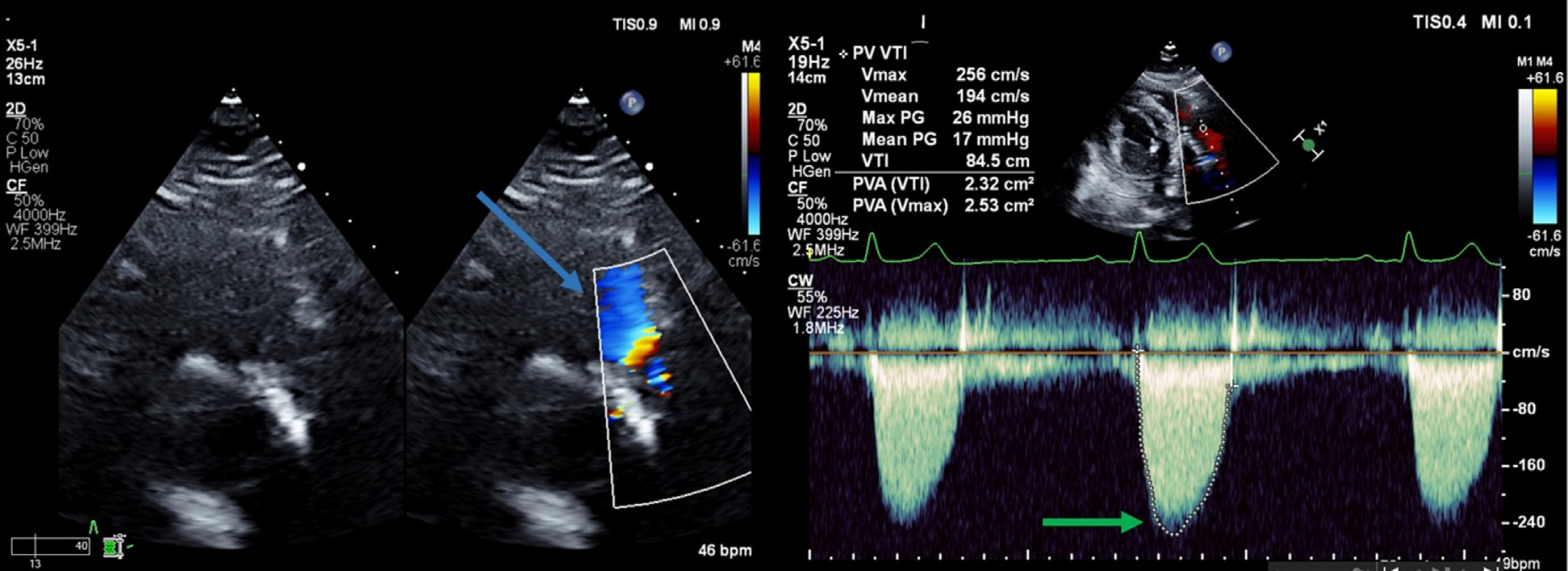
Transthoracic Echocardiogram Imaging uses color Doppler to highlight flow acceleration during systole, marked by the blue arrow. It also features a continuous wave (CW) Doppler analysis of a prosthetic pulmonary valve. The mean gradient across the valve, which is indicated by the green arrow, is measured at 17 mm Hg. CF = color flow; max = maximum; PG = pressure gradient; PV = pulmonary valve; PVA = pulmonary valve area; V = velocity, VTI = velocity time integral; 2D = 2-dimensional.

In 2023, she developed dyspnea, and TTE revealed pulmonic stenosis, with a mean gradient of 17 mm Hg across the pulmonary valve (Figure 2, Video 2), an increase from a previous mean gradient of 6.1 mm Hg, and demonstrated moderate hemodynamic valve deterioration on the basis of Valve Academic Research Consortium 3 criteria.^1^ We proceeded with imaging of the pulmonary valve using cardiac CTA to evaluate for valve degeneration vs pannus formation vs thrombosis.

**Figure 3:**
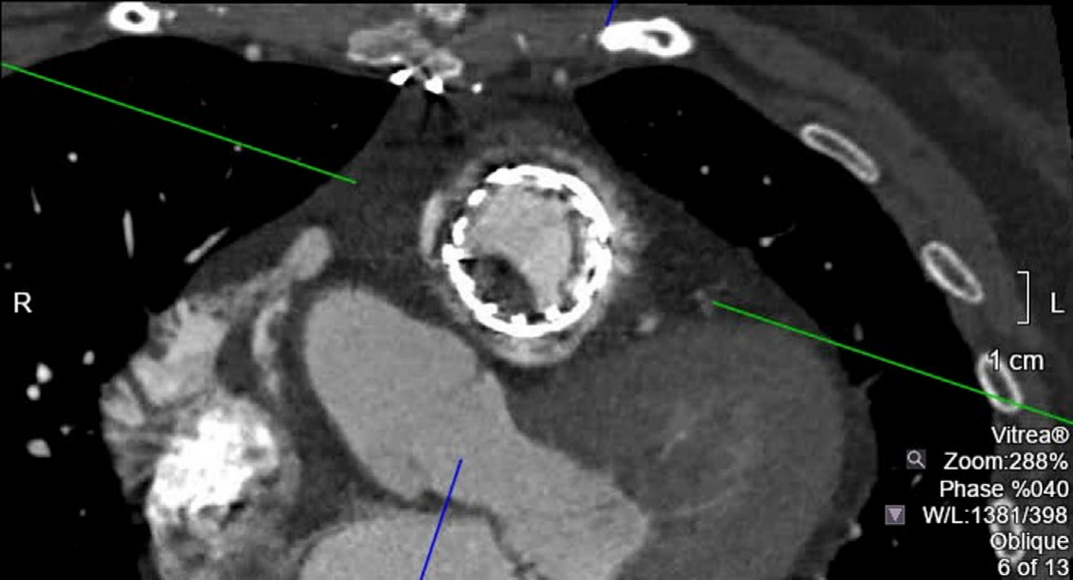
Short-Axis View of the Prosthetic Pulmonary Valve Imaging shows valve thrombosis involving the inferior-posterior leaflet. L = left; R = right.

**Figure 4:**
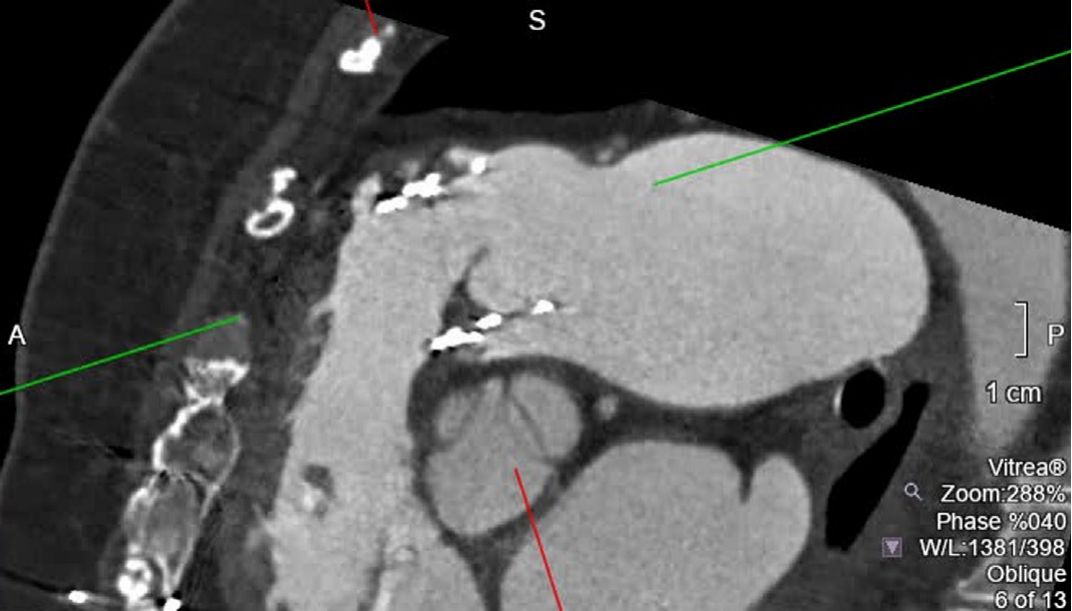
Long-Axis View, Prosthetic Pulmonary Valve Imaging shows valve thrombosis involving the inferior-posterior leaflet. A = anterior; P = posterior; S = superior.

**Figure 5:**
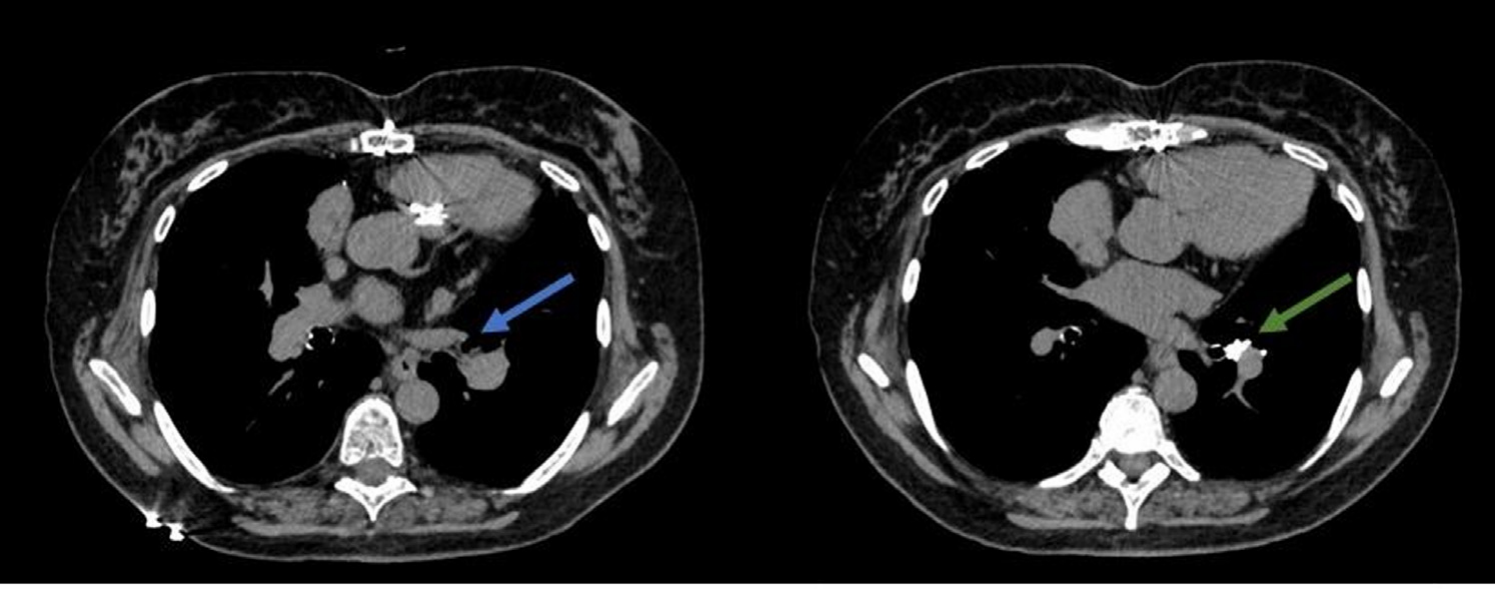
Computed Tomography Angiography of the Chest Imaging demonstrates significant enlargement of the central pulmonary arteries, consistent with chronic pulmonary valve disease. Additionally, a calcified subocclusive chronic thrombus is identified in the left lower lobe pulmonary artery. This finding, indicated by the blue and green arrows, highlights the location of the thrombus at the origin of the segmental arterial branches of the basilar segments of the left lower lobe.

CTA of the chest revealed valve thrombosis along the stent of the pulmonary valve (Figures 3 and 4, Videos 4 and 5). This finding indicates that the prosthesis is thrombosed and obstructed. She was also noted to have a pulmonary artery thrombus at the origin of the segmental arterial branches of the basal segments of the left lower lobe. Additionally, CTA showed enlargement of the main pulmonary arteries, attributed to the long-standing effects of chronic pulmonary valve disease (Figure 5). She received anticoagulation therapy with warfarin, and her international normalized ratio was maintained between 2 and 3.

## Management

The most severe risk associated with valve thrombosis is the development of embolization. In cases of obstructive valve thrombosis, this could lead to embolization, potentially causing a catastrophic pulmonary embolism, depending on the size of the clot.^2^ Our patient experienced a small branch pulmonary artery embolism. Thus, management of obstructive valve thrombosis typically involves anticoagulation therapy with heparin and warfarin to prevent further thrombus formation and promote valve function. In many instances, anticoagulation is associated with lower rates of thrombosis development compared with placebo.^3^

This case can be classified as obstructive valve thrombosis given the presence of symptoms, thrombosis in the pulmonary artery, and an increase in gradient. HALT is typically defined as subclinical computed tomography (CT) findings and is a well-described phenomenon following transcatheter aortic valve replacement (TAVR). However, this is the first documented case report of valve thrombosis involving the pulmonary valve-in-valve. Evidence-based treatments are noted to involve TAVR devices. Although trials with direct oral anticoagulants have begun and are ongoing, the GALILEO trial (Global Study Comparing a rivAroxaban-based Antithrombotic Strategy to an antipLatelet-based Strategy After Transcatheter aortIc vaLve rEplacement to Optimize Clinical Outcomes) for rivaroxaban illustrated a decreased incidence of HALT and valve thrombosis compared with antiplatelet therapy but an increased risk of all-cause mortality and major bleeding events.^4^^,^^5^ The optimal duration of anticoagulation for valve thrombosis and the potential need for valve replacement should be individualized on the basis of patient characteristics, valve type, and clinical response. When anticoagulation is contraindicated or ineffective, surgical valve replacement may be considered as a definitive treatment option.

## Discussion

Valve thrombosis is a phenomenon primarily associated with bioprosthetic heart valves that is increasingly recognized.^6^ It is characterized by abnormal thickening of valve leaflets with low attenuation, observable on CT imaging, and is often attributed to thrombus formation. The precise cause of valve thrombosis remains incompletely understood, but turbulent blood flow, endothelial damage, and changes in blood composition are considered contributing factors.^7^ Clinically, obstructive valve thrombosis is significant because it can lead to valve dysfunction, thromboembolic events, and heart failure.

Although valve thrombosis has predominantly been described in bioprosthetic aortic valves, its occurrence in bioprosthetic pulmonary valves is exceedingly rare, as demonstrated in this report. The clinical implications of valve thrombosis for our patient were significant. CTA revealed obstructive valve thrombosis along the pulmonary valve stent, with thrombus formation. Furthermore, enlargement of the central pulmonary arteries observed on CTA was consistent with chronic pulmonary valve disease, thus emphasizing the importance of timely diagnosis and management.

This case represents the first documented instance of valve thrombosis following a TPVR valve-in-valve procedure. It raises the possibility that with the increased introduction and rising use of transcatheter devices for right-sided cardiac interventions, we could see a corresponding increase in the incidence of HALT or valve thrombosis associated with these devices. A particular theoretical concern is that the increase in valve-in-valve procedures using transcatheter methods could increase the risk of blood stasis, thereby altering laminar flow and potentially leading to obstructive valve thrombosis on the surfaces of these devices. Although no direct causal relationships have been definitively established to date, the potential connection warrants further investigation. This case underscores the urgent need for more comprehensive research into the mechanisms and prevention of valve thrombosis, particularly in the context of advanced transcatheter technologies.

## Follow-Up

**Figure 6:**
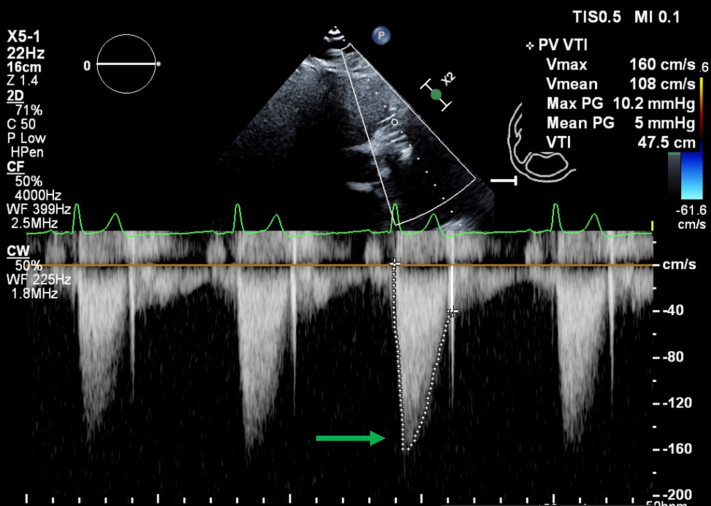
Transthoracic Echocardiogram After 4 Weeks of Anticoagulation Imaging demonstrates a continuous-wave (CW) Doppler measurement of the mean gradient, indicated by the green arrow, of 5 mm Hg. Abbreviations as in Figure 2.

**Figure 7:**
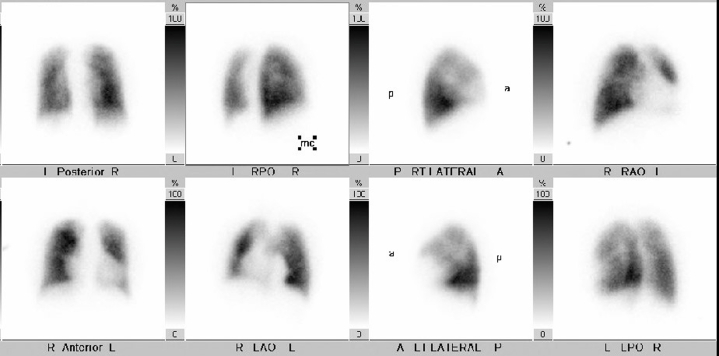
Nuclear Medicine Ventilation-Perfusion Scan Imaging shows perfusion images with mild inhomogeneity, indicating uneven distribution of blood flow in the lungs, but without any significant mismatched perfusion defects, which would suggest that areas of the lung are well ventilated. The single-breath ventilation image appears normal, and the washout phase is also normal, indicating effective clearance of air from the lungs without obstruction or retention. No evidence of pulmonary embolism is noted. I = inferior; LAO = left anterior oblique; LPO = left posterior oblique; LT = left; RAO = right anterior oblique; RPO = right posterior oblique; RT = right; other abbreviations as in Figures 3 and 4.

In the subsequent months, the patient reported mild improvement in her dyspnea symptoms and lower extremity edema, necessitating the use of intermittent diuretic therapy. With the administration of warfarin, her most recent TTE showed a mean gradient of 5 mm Hg, which suggests a reduction in thrombus burden (Figure 6, Video 3). She will remain on lifelong anticoagulation with TTE follow-up. Given her elevated creatinine level, she underwent a nuclear medicine ventilation-perfusion scan 3 months later for the evaluation of pulmonary embolism. The scan demonstrated no evidence of pulmonary embolism (Figure 7).

## Conclusions

Obstructive valve thromboses are clinically significant complications in patients with bioprosthetic heart valves. This case report highlights the first reported instance of obstructive valve thrombosis in a bioprosthetic pulmonary valve and underscores the importance of early recognition, multimodal imaging for diagnosis, and individualized treatment strategies. Although anticoagulation therapy is often the first-line approach, surgical valve replacement may be necessary in patients with persistent valve dysfunction or contraindications to anticoagulation. Further research is needed to understand the pathophysiology of valve thrombosis more clearly and to refine treatment strategies for this condition.

## Funding Support and Author Disclosures

The authors have reported that they have no relationships relevant to the contents of this paper to disclose.

## Appendix

Video 1 Transthoracic Echocardiogram in the Right Ventricular Inflow Tract View Imaging reveals a well-seated bioprosthetic pulmonary valve, exhibiting normal structural integrity. The Doppler flow study demonstrates a normal valve gradient, indicating unobstructed blood flow through the valve, with no evidence of stenosis or regurgitation.

Video 2 Transthoracic Echocardiogram in the Right Ventricular Inflow Tract View Imaging reveals a well-seated bioprosthetic pulmonary valve. The Doppler flow study demonstrates an elevated gradient, suggesting increased resistance to blood flow across the valve.

Video 3 Transthoracic Echocardiogram in the Right Ventricular Inflow Tract View Imaging reveals a well-seated bioprosthetic pulmonary valve. The Doppler flow study demonstrates a reduced gradient from previously, suggesting an improvement in valve function and indicating a resolution in valve thrombosis.

Video 4 Axial Cine Video From the Computed Tomography Angiography Scan Imaging reveals a thrombosis along the bioprosthetic valve. The thrombus is prominently observed along the inferior-posterior leaflet.

Video 5 Sagittal Cine Video From the Computed Tomography Angiography Scan Imaging further corroborates the presence of a thrombus on the bioprosthetic valve. The thrombus is located along the inferior-posterior leaflet, mirroring the findings in the axial view.
